# Effects of Immobilized Ionic Liquid on Properties of Biodegradable Polycaprolactone/LDH Nanocomposites Prepared by In Situ Polymerization and Melt-Blending Techniques

**DOI:** 10.3390/nano10050969

**Published:** 2020-05-18

**Authors:** Sonia Bujok, Jiří Hodan, Hynek Beneš

**Affiliations:** Institute of Macromolecular Chemistry, CAS, Heyrovského nám. 2, 162 06 Prague 6-Břevnov, Czech Republic; bujok@imc.cas.cz (S.B.); hodan@imc.cas.cz (J.H.)

**Keywords:** polycaprolactone nanocomposites, layered double hydroxides, microwave-assisted polymerization, ionic liquid

## Abstract

The high capacity of calcinated layered double hydroxides (LDH) to immobilize various active molecules together with their inherent gas/vapor impermeability make these nanoparticles highly promising to be applied as nanofillers for biodegradable polyester packaging. Herein, trihexyl(tetradecyl)phosphonium decanoate ionic liquid (IL) was immobilized on the surface of calcinated LDH. Thus, the synthesized nanoparticles were used for the preparation of polycaprolactone (PCL)/LDH nanocomposites. Two different methods of nanocomposite preparation were used and compared: microwave-assisted in situ ring opening polymerization (ROP) of *ε*-caprolactone (*ε*CL) and melt-blending. The in situ ROP of *ε*CL in the presence of LDH nanoparticles with the immobilized IL led to homogenous nanofiller dispersion in the PCL matrix promoting formation of large PCL crystallites, which resulted in the improved mechanical, thermal and gas/water vapor barrier properties of the final nanocomposite. The surface-bonded IL thus acted as nanofiller surfactant, compatibilizer, as well as thermal stabilizer of the PCL/LDH nanocomposites. Contrary to that, the melt-blending caused a partial degradation of the immobilized IL and led to the production of PCL nanocomposites with a heterogenous nanofiller dispersion having inferior mechanical and gas/water vapor barrier properties.

## 1. Introduction

Biologically degradable polyesters have attracted increasing attention due to their non-toxicity, biocompatibility and low impact on the environment, which makes them attractive for food packaging, medicine, agriculture, etc. [1]. However, pure polyesters generally exhibit poor barrier performance, which limits their widespread use. Therefore, recent research has been focused on investigation of polyester nanocomposites prepared by ring opening polymerization (ROP) of cyclic monomers such as *ε-*caprolactone (*ε*CL), lactide (LA), glycolide (GA), and their copolymers, or condensation copolyesters (e.g. poly(butylene adipate-co-terephthalate, PBAT) with natural or synthetic clays like montmorillonite (MMT), hydrotalcite or layered double hydroxides (LDH) [1,2,3,4]. In comparison with conventional polymer composites, the small content of nanoclays insignificantly or only slightly alters processing parameters (e.g., viscosity, density, melting, and glass transition temperature), but efficiently improves the mechanical, flame retardant, and barrier properties of final polymer materials [5].

LDH represents a group of 2D anionic clays analogical to brucite-like materials (e.g., hydrotalcite) described by a formula [M^2+^_1−*x*_M^3+^*_x_*(OH)_2_]*^x^*^+^[A*^n^*^−^*_x_*_/*n*_]∙mH_2_O, where M^2+^ is a divalent cation (e.g., Ca^2+^, Mg^2+^, Cu^2+^, Zn^2+^), M^3+^ is a trivalent cation (e.g., Al^3+^, Fe^3+^, La^3+^), and the value of *x* (in the range 0.2–0.33) refers to the M^2+^/M^3+^ = (1−*x*)/*x* molar ratio. Two surface hydroxyl groups are coordinated by each metal cation forming a positively charged sheet. In order to compensate for the positive charge of the layers, exchangeable anions (A*^n^*^−^, e.g., CO_3_^2−^, NO_3_^−^, Cl^−^) and molecules of water are intercalated into LDH galleries [6,7]. Contrary to natural clays like montmorillonite and kaolinite, the use of LDH in polymer nanocomposites is particularly advantageous because of variable and tunable LDH structure. LDH can thus be tailor-made for immobilization of e.g., catalytically active metals, metal oxides or metal complexes, UV sensitizers, surfactants, ionic liquids or organometallic complexes [8,9,10]. The application of LDH with immobilized active compounds in polymers can then present an interesting strategy to prepare functional nanocomposites used for, e.g., smart packaging sensitive to external stimuli (humidity, temperature, pH, etc.), antibacterial packaging for foods, etc. [11,12,13]. Moreover, recent research has shown that LDH adsorption capacity can be strongly enhanced by calcination process, thus calcinated form of LDH indicate improved sorption performance of organic species, e.g., dyes [14,15]. Therefore, the high capacity of calcinated LDH to immobilize molecules together with the inherent impermeability for gasses and vapors make them promising nanoscale fillers for biodegradable polyester packaging.

Appropriate barrier properties have become increasingly important in protective coatings and packaging applications in food industry, pharmaceuticals and electronic devices [5]. Gas and water vapor barrier properties of nanocomposites are mainly influenced by lateral dimension, orientation, dispersion and degree of exfoliation of lamellar nanofillers in polymer matrix [5]. It is extremely important to reach homogeneous dispersion of nanoclays and high degree of their exfoliation, because large numbers of individually dispersed nanosheets form mechanical barriers decreasing overall diffusion of gas/vapor molecules by creating “a torturous path” in nanostructured materials [5].

Desired homogeneous nanofiller dispersion and exfoliation are challenging issues due to the incompatibility between hydrophobic polymer matrix and hydrophilic clay. Therefore, proper organophilization of clays is the most important step to improve polymer–nanofiller compatibility and to achieve a homogenous morphology of nanocomposites.

However, final nanocomposite morphology is also influenced by preparation method. In general, three main ways are used for manufacturing of polymer/clay nanocomposites. The first approach is melt-intercalation (melt-blending), in which polymer chains in molten state can be directly intercalated into interlayer space. This method is a solvent-free process but requires high shear forces and good affinity between organoclay and polymer, which promotes peeling single nanoplatelets from tactoids [16].

The second technique is solution intercalation, in which a polymer is first dissolved in a suitable solvent, then mixed with the clay suspension under vigorous stirring or sonication and finally the solvent is evaporated. This approach is generally not preferred, because it is more complex, environmentally unfriendly and limited to specific polymer/solvent pairs.

The third way is in situ polymerization, which involves polymerization in the presence of clay dispersed in a liquid monomer. The use of cyclic monomers (esters), which polymerize via in situ ROP, is highly convenient, because no solvent is necessary [17,18]. Small molecules of monomers penetrate into interlayers, which induces polymerization within the clay galleries. The growing polymer chain then forces clay delamination. The clay delamination can be even more intensive when ionic liquids (IL)s are used as organic modifiers and when the IL-modified LDH are exposed to microwave irradiation [18,19].

Due to low volatility, good chemical and thermal stability, as well as non-flammability, ILs have become popular additives for various polymer nanomaterials, especially for nanocomposites, in which they are currently used as solvents, surfactants, coupling agents and compatibilizers [20]. Moreover, it has been reported that ILs immobilized on the LDH surface exhibited catalytic-initiating effect on ROP of *ε*CL [18,19]. Water molecules from LDH (surface-adsorbed or intercalated) initiate ROP of *ε*CL, while IL-anions catalyze a polymerization reaction [19]. The higher water content in LDH means the higher number of active centers, which results in decreased molecular weight of the produced polycaprolactone (PCL). Therefore, in order to produce high molecular weight PCL, the amount of water in LDH nanoparticles must be lowered by e.g., calcination.

In this work, we have prepared a low-water content LDH by calcination of Ca/Al LDH with subsequent surface modification with trihexyl(tetradecyl)phosphonium decanoate IL (IL-D). The prepared IL-functionalized LDH has been tested for preparation of PCL/LDH nanocomposite films using two different approaches: melt blending and in situ ROP of *ε*CL in the presence of calcinated LDH. The effects of immobilized IL-D on morphology, thermal, mechanical, and gas/vapor properties of final PCL/LDH nanocomposite films have been studied.

## 2. Materials and Methods

### 2.1. Materials

**Figure 1:**
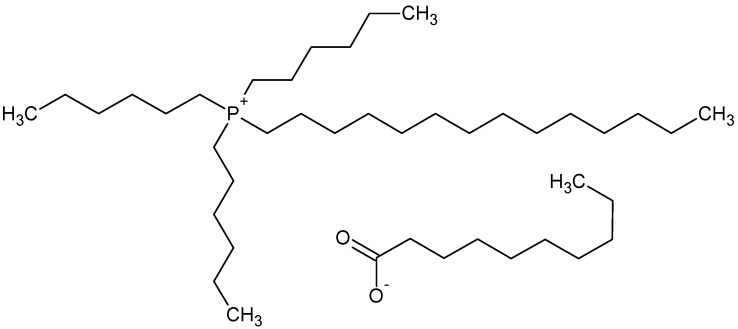
Chemical structure of trihexyl(tetradecyl)phosphonium decanoate (IL-D).

Aluminium nitrate nonahydrate (Al(NO_3_)_3_), calcium nitrate tetrahydrate (Ca(NO_3_)_2_), sodium nitrate, sodium hydroxide, ethanol (p.a.), and tetrahydrofuran (THF) (p.a.) were supplied by Lach-Ner (Neratovice, Czech Republic) and used as received. Trihexyl(tetradecyl)phosphonium decanoate IL (IL-D, Figure 1) provided by IoLiTec (Heilbronn, Germany) was used without further purification.

*ε*CL and tin(II) 2-ethylhexanoate (catalyst) were supplied by Sigma-Aldrich (Prague, Czech Republic). Monomer (*ε*CL) was distilled under reduced pressure (over CaH_2_) and stored under inert (nitrogen) atmosphere. Commercial PCL Capa 6800 was kindly provided by Perstorp AB (Malmö, Sweden).

### 2.2. Modification of Calcinated Ca^2+^/Al^3+^ Layered Double Hydroxide with Phosphonium-Based IL

Pristine LDH (Ca/Al) was synthesized from Ca(NO_3_)_2_, Al(NO_3_)_3_, NaOH and NaNO_3_ by the co-precipitation method reported in our previous work [19]. The pristine LDH was heated at 500 °C (24 h) to receive a calcinated LDH (C-Ca/Al). Then, C-Ca/Al was modified by IL-D (2.5 g) solution in dry THF (75 mL THF) using the ratio of 1 g C-Ca/Al per 100 mL of dry THF at 60 °C for 24 h under inert (N_2_). The sludge was then filtered under reduced pressure, washed with THF and finally dried (80 °C for 12 h). The received product (C-Ca/Al-D) was freshly dried (100 °C, 7 h in a vacuum) directly before experiments.

### 2.3. Ring Opening Polymerization of ε-Caprolactone in the Presence of LDH

Mixture of *ε*CL, catalyst and LDH (1.0 wt %) was placed into a closed reaction vessel (10 mL) and immersed into ultrasound bath for 10 min. Then, the vessel was placed into the monomode microwave reactor (CEM Discover SP, *f* = 2.45 GHz), heated to 120 °C and kept at this temperature using constant temperature mode. The vessel was cooled down after given time in the microwave reactor (to 100 °C) and then in a dry ice/ethanol bath. For further analyses and testing, PCL-LDH nanocomposites were prepared as films of thickness 0.2 mm (compression molding using PTFE mold at 120 °C with 50 and 100 kN compression for 2 min each).

### 2.4. PCL-LDH Nanocomposites Preparation by Melt-Blending

PCL-LDH nanocomposites based on Capa 6800 and LDH were prepared using Brabender plasticorder with mixing chamber B50 EHT (Brabender, Duisburg, Germany) by mixing at 120 °C for 10 min at rotation speed 60 rpm. For further analyses and testing, PCL-LDH nanocomposites were prepared as films of thickness 0.2 mm (compression molding using PTFE mold at 120 °C with 50 and 100 kN compression for 2 min each).

### 2.5. Characterization Methods

Attenuated total reflectance technique (ATR-FTIR) was used to measure infrared spectra of the prepared LDH samples using Spectrum 100 T FT-IR spectrometer (Perkin Elmer, Waltham, MA, USA) equipped with DTSG detector and a universal ATR accessory with diamond/ZnSe crystal. FTIR spectra were measured in the range of 650–4000 cm^−1^ as average of 16 scans per spectrum using a resolution of 4 cm^−1^.

The X-ray Diffraction (XRD) pattern of Ca/Al sample was recorded on a high-resolution diffractometer Explorer (GNR Analytical Instruments, G.N.R. S.r.l., Agrate Conturbia, Italy) equipped with a 1 D silicon strip detector Mythen 1 K (Dectris, Baden-Daettwil, Switzerland) was used. Sealed X-ray tube (operated at 40 kV and 30 mA) monochromatized with Ni foil (β filter) was used as a source of CuKα radiation (wavelength λ= 1.54 Å). The measurements were performed in Bragg-Brentano geometry (*2θ* = 1°–50° with a step 0.1°) with the exposure time of 15 s at each step.

Wide angle X-ray scattering (WAXS) patterns of C-Ca/Al and C-Ca/Al-D samples were recorded on a pinhole camera (MolMet, Rigaku, Tokyo, Japan, modified by SAXSLAB/Xenoxs) attached to a microfocused X-ray beam generator (Rigaku MicroMax 003) which operates at 0.6 mA and 50 kV. The camera was equipped with a Pilatus 300 K detector (vacuum version). The experimental setup covered a *q* range of 0.145–3.45 Å^−1^ (*2θ* = 2°–50°) with *q* defined as *q* = 4π/λ·sin*θ* (λ–wavelength, 2*θ*-scattering angle). Each measurement had the exposure time of 3600 s. The calibration of sample-to-detector distance was done using a silver behenate powder.

The morphology of the prepared LDH nanoparticles was observed on scanning electron microscope (SEM) MAIA3 (Tescan, Brno, Czech Republic) using high vacuum mode (5 kV). Samples were first dispersed in ethanol and treated with ultrasound for 3 min and then were dropped on the fresh mica surface and left to completely dry (at RT). Before SEM experiments, the sample surface was covered by a conductive thin platinum film using vacuum sputter coater: SCD050 (Balzers), SCD 050 (Leica).

Thermogravimetric analysis (TGA) of LDH and PCL-LDH nanocomposites was performed on Pyris 1 TGA (Perkin Elmer) at heating rate of 10 °C/min from 30 °C to 750 °C and from 30 °C to 600 °C, respectively, at nitrogen atmosphere (25 mL/min).

Differential scanning calorimetry (DSC) was used for determination of the melting onset temperature (*T*_m_) and crystallinity (*X*_c_) of the PCL/LDH nanocomposite films using the Q2000 calorimeter (TA Instruments, New Castle, DE, USA) in heating-cooling-heating mode in the temperature range between −80°C and 250 °C at a constant heating rate 10 °C·min^−1^ under inert atmosphere (nitrogen purge flow of 50 cm^3^/min).

Dynamic-mechanical and thermal analyses (DMTA) of PCL-LDH nanocomposites were performed on ARES-G2 rheometer (TA Instruments, USA). The temperature dependence of complex shear modulus (G*) was determined on rectangular samples (20 × 10 × 2 mm) using oscillatory shear deformation (0.4% strain) at 1 Hz frequency from −100 °C to 60 °C at temperature ramp rate of 3 °C·min^−1^. The main transition temperature (*T*_α_) was determined as the tan *δ* (loss tangent) peak maximum (the precision of the measurements was *T*_α_ ± 2 °C).

The number average molecular weight (*M*_n_) and dispersity (*Đ*) of PCL/LDH nanocomposites were determined using a gel permeation chromatography (GPC) system equipped with refractive index detector Shodex RI-101 ( Showa Denko, Kawasaki, Japan), UV-Vis detector (λ = 254 nm, LCD 2084, Ecom, Prague, Czech Republic) and set of three PLgel columns (pore size: 50/10E3/10E4 Å, 300 × 7.5 mm, particle size of 10 µm, Agilent, Santa Clara, CA, USA). Eluent (tetrahydrofuran) was purged with a 1 mL/min flow rate. Polystyrene standards were used for calibration.

Contact angle measurements were performed by the sessile drop technique using Contact angle system OCA (DataPhysics) by placing 5 µL droplet of distilled water on the surface of PCL/LDH film. Droplets were analyzed and contact angle (*θ_H2O_*) was determined using SCA20 (DataPhysics) software by Laplace-Young fitting.

Unaxial tensile tests of PCL/LDH nanocomposite films were performed on Instron machine 6025/5800 R (Instron Limited, Norwood, MA, USA) equipped with a 100 N load cell at RT using 50 mm/min cross-head speed. Five specimens of each nanocomposite in the shape of a dumbbell (ISO 527-3/5, half size), with a length of 60 mm and thickness of 0.2 mm, were used for tensile test.

Gas transport properties of PCL/LDH nanocomposite films were investigated by time-lag permeation method [21]. Thin films (2–3 samples of each nanocomposite membrane) were inserted into a membrane cell which was then placed into permeation apparatus. Samples were then evacuated (vacuum of 10^−4^ mbar) for 6 h at 30 °C. Feed pressure *p*_i_ was 1.5 bar. The permeability coefficient (*P*) was calculated from the increase of the permeate pressure (Δ*p_p_*) per time interval (Δ*t*) in a calibrated volume (*V_p_*) of the product part during the steady state of permeation. For calculation of the permeability coefficient, Equation (1) was used:

*P* = (Δ*p_p_*/Δ*t*)·(*V_p_l*/*Ap_i_*)·(1/*RT*) (1)

where *l* represents membrane thickness, *A* the area, *T* the temperature and *R* the ideal gas constant. Permeabilities were reported in units of Barrer (1 Barrer =10^−10^ cm^3^ (STP)·cm/(cm^2^·s·cmHg) = 3.3539 × 10^−16^ mol·s^−1^·m^−1^·Pa^−1^). Relative standard deviation (SD) of permeability coefficient measurement was 2.4%. SD for water vapor measurement was close to 10% because feed pressure for water vapor was much lower than that for gases (and was in the bottom of the pressure transducer span with a higher error of measurement).

Gas diffusivities (*D*) were determined from the time-lag data, using the following equation:

*D* = *l*^2^/6*θ* (2)

where *l* is the thickness of membrane and *θ* is the time-lag. Relative standard deviation of diffusion coefficients was 4%. Apparent solubility coefficients (*S*) were determined using Equation (3):

*S* = *P*/*D* (3)

The ideal separation factor (*α_ij_*) defines the overall selectivity of membrane for a pair of gases (*i* and *j*) according to:

*α_ij_* = *P_i_*/*P_j_* = (*S_i_*/*S_j_*)·(*D_i_*/*D_j_*) (4)

where *P_i_*/*P_j_* are pure gas permeabilities, *S_i_*/*S_j_* and *D_i_*/*D_j_* are the solubility and the diffusion selectivity, respectively.

## 3. Results and Discussion

### 3.1. Preparation of IL-Functionalized LDH Nanoparticles with Low-Water Content

According to our latest paper, IL-modified Ca^2+^/Al^3+^ LDH nanoparticles can be considered as a functional nanofiller with catalytic/initiating effect on ROP of *ε*CL, which enables synthesis of organometallic catalyst-free PCL [19]. Since the *ε*CL polymerization in the presence of IL-functionalized LDH is initiated by adsorbed/interlayer water, the total amount of water in LDH must be lowered in order to produce a high molecular weight PCL. The content of water in the IL-modified LDH can be minimized by vacuum drying to ca. 10 wt %, which still is not sufficient; e.g., applying 5.0 wt % of the vacuum-dried LDH for in situ *ε*CL ROP produces PCL with M_n_ of only ca. 3000 g/mol [19]. The low molecular weight PCL does not allow us to prepare mechanically robust nanocomposite films, suitable for application testing (mechanical and barrier properties).

**Figure 2:**
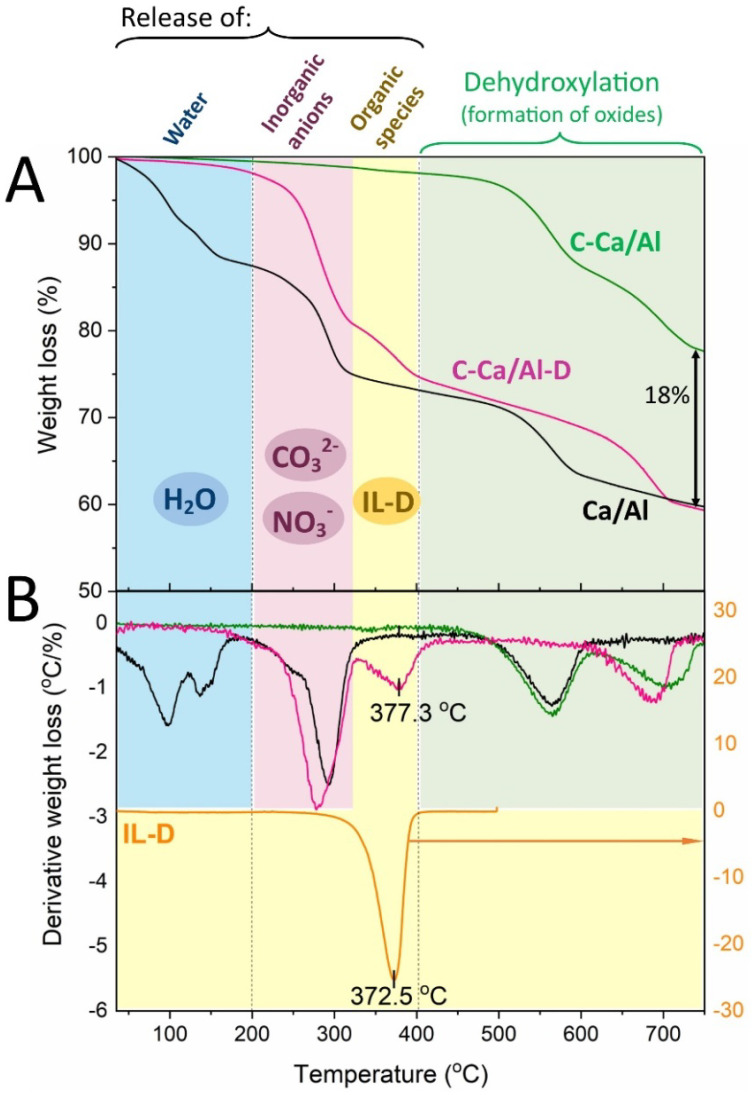
TGA (**A**) and DTG (**B**) curves of pristine LDH (Ca/Al), calcinated LDH (C-Ca/Al), calcinated LDH modified with IL-D (C-Ca/Al-D) and pure ionic liquid (IL-D).

As a consequence, thermal treatment of pristine LDH was a necessary step, which led to production of less hydrophilic calcinated LDH (C-Ca/Al) with much lower water content in comparison to the pristine LDH (Ca/Al) (Figure 2). The TGA/DTG curves of the calcinated LDH (C-Ca/Al) clearly show the decreased content of adsorbed/intercalated water, which is released during the first weight loss (<200 °C). The calcination process advantageously increases the thermal stability of C-Ca/Al particles, which are thermally stable up to ca. 450 °C.

Subsequent organic modification of C-Ca/Al with IL-D enabled to produce IL-functionalized LDH (C-Ca/Al-D) with the content of water around 1.5 wt % (Figure 2). In contrast to C-Ca/Al, the TGA/DTG curves of IL-modified LDH (C-Ca/Al-D) reveal additional decomposition step in the temperature range of 200–400 °C, which is related to the presence of surface-bonded IL (pure IL decomposes at ca. 370 °C). The overall IL content in C-Ca/Al-D was equal to 18 wt %, as it was estimated from the difference in total weight loss between C-Ca/Al and C-Ca/Al-D [19,22].

The successful immobilization of the IL molecules on the C-Ca/Al-D surface was further confirmed by FTIR spectroscopy (Figure S1A) showing the presence of C–H (2850–2960 cm^−1^) and –(C=O)O– (1540–1570 cm^−1^) stretching vibrations. The FTIR spectra of C-Ca/Al and C-Ca/Al-D also indicate vanishing of the bending vibrations at 1640 cm^−1^ characteristic for water molecules, which proves (in accordance to the TGA results) the decreased water content after calcination.

Due to chemical composition of the pristine Ca/Al LDH (Ca^2+^/Al^3+^ cations and NO_3_^−^/CO_3_^2−^ interlayer anions), the calcination process leads to collapse of the layered structure and formation of 2D crystals of calcite as the most stable polymorph of calcium carbonate [23]. Thus, the produced calcinated LDH (C-Ca/Al) is typically composed of calcium carbonate and traces of mixed metal oxides [23,24,25]. IL-functionalization did not affect the crystalline structure of the calcinated LDH, as indicated from the crystallographic XRD/WAXS patterns (Figure S1B) of C-Ca/Al (green curve) and IL-functionalized C-Ca/Al-D (pink curve) showing highly crystalline structure containing typical reflections of calcite crystallites [25]. The SEM microphotographs (Figure S1C) reveal a hexagonal structure of the calcinated LDH with lateral dimensions ranging in the units of micrometers.

### 3.2. PCL-LDH Nanocomposites: In Situ ROP vs. Melt-Blending

Both prepared IL-functionalized (C-C/Al-D) and non-functionalized (C-Ca/Al) LDH nanoparticles have been further applied as the nanofiller (1.0 wt %) in PCL matrix via (i) the in situ ROP of *ε*CL under microwave irradiation and (ii) the melt-blending (Table 1). The nanofiller content of 1.0 wt % was selected as optimum based on the appropriate initiating-catalytic activity of IL-functionalized LDH nanoparticles during the in situ ROP of *ε*CL (more details can be found in our last study [19]). Other studies also report that 1 wt % addition of LDH into PCL matrix is optimal and leads to homogenously dispersed LDH nanoparticles with no formation of agglomerates [26]. Relatively fast, simple, and solvent-free melt blending has been chosen as the comparative preparation technique of the PCL/LDH nanocomposites.

**Figure 3:**
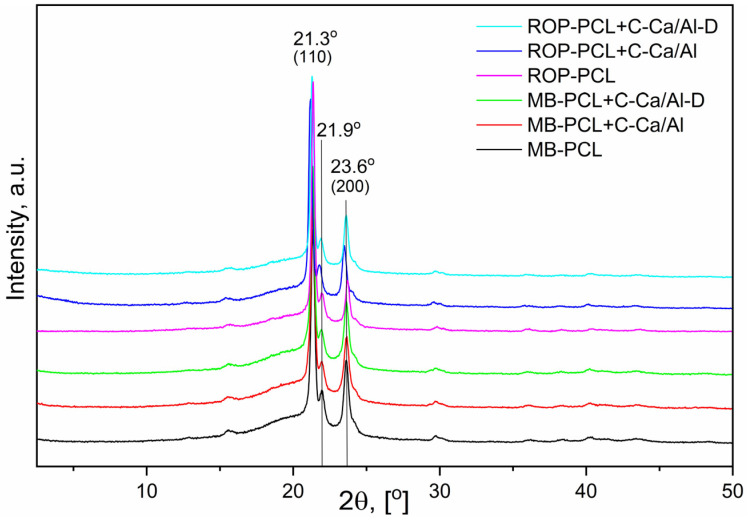
XRD patterns of prepared PCL-LDH nanocomposites.

Crystalline structure of PCL-LDH nanocomposites was investigated using X-ray diffraction method. XRD patterns of all PCL/LDH nanocomposite and neat PCL films (Figure 3) indicate the presence of PCL crystalline fraction confirmed by the reflections at 2θ = 21.3°, 21.9° and 23.6°, which are characteristic for PCL matrix [26]. No signals related to calcinated LDH nanofillers appeared on the XRD patterns.

The influence of the LDH type and preparation method on the sizes of the PCL crystallites was studied using Scherrer Equation (5):

*D_(hkl)_* = *Kλ*/*βcosθ* (5)

where *K* is shape factor (*K* = 0.9), *λ* is X-ray wavelength, *β* is FWHM of the *hkl* reflection peak corresponding to crystalline domain, *θ* is the Bragg angle related to the *hkl* reflection. Sizes of the crystalline domains *D*_(110)_ present in all PCL-LDH nanocomposite films are listed in Table 2. Addition of the LDH nanoparticles strongly affects crystallite formation regardless the preparation method. Crystalline domains present in the PCL/LDH nanocomposite films are ca. 2.2–2.8 times larger than those of neat PCL. It indicates that nanofillers have nucleating effect on PCL crystallization [27]. The largest PCL crystalline domains were observed in the in situ prepared nanocomposite film containing IL-functionalized LDH (ROP-PCL + C-Ca/Al-D). It was reported in our previous study [19] that microwave irradiation of IL-functionalized LDH promotes their delamination and exfoliation in the PCL matrix. LDH nanoparticles are then homogenously distributed and well-separated to single uniform LDH nanoplatelets. Their nucleating efficiency is thus much more improved in comparison to large and worse-dispersed agglomerates of non-modified LDH [28].

The crystallization behavior of PLC/LDH nanocomposite films was further characterized by DSC (Figure S2). Melting temperature (*T_m_*), melting enthalpy (Δ*H_m_*) and degree of crystallinity (*X_c_*) were evaluated (Table 2). In case of melt-blending, the addition of non-modified C-Ca/Al particles did not affect the nanocomposite crystallinity (42.8%) in comparison to the neat PCL (42.3%). Contrary to that, the crystallinity of melt-blended nanocomposite with IL-functionalized fillers (C-Ca/Al-D) slightly increased (46.2%) confirming better dispersion of LDH particles and its nucleating effect. In the case of in situ ROP of *ε*CL, the crystallinity of synthesized nanocomposites is affected by the addition of the nanoparticles as well as the molecular weight of the in situ formed PCL (Table 3).

The DMTA results (Table 2 and Figure S3) show that the main transition temperature (*T_α_*) of all prepared PLC/LDH nanocomposite films is similar to the neat PCL.

Thermal stability of the prepared materials was evaluated using TGA (Table 2, Figure S4). It is well known that the presence of LDH can accelerate the degradation of PCL matrix [26]. However, in the case of ROP-PCL nanocomposites series, opposite phenomenon was observed. Temperature at 5 wt % of total sample weight loss (*T_d5%_*) for the samples containing LDH (IL-modified and non-modified) increased in comparison to neat PCL regardless significantly lower molecular weight of both ROP-synthesized nanocomposites (Table 3). Generally, the thermal degradation of polymer composites is affected by various factors, e.g., molecular weight, morphology, and crystallinity of the polymer matrix, as well as the presence and type of the filler and used compatibilizer [29], e.g., the use of highly hydrophilic clays promotes thermally-induced hydrolysis of aliphatic polyester-clay nanocomposites [30]. In our case, the more hydrophobic calcinated LDH nanoparticles with low content of water and no layer-coordinated hydroxyl groups were used and therefore their addition improved thermal stability of the nanocomposites of the ROP-PCL series. The slightly higher *T_d5%_* of ROP-PCL + C-Ca/Al than ROP-PCL can be also related to 2.2-fold larger PCL crystallites formed in the sample containing C-Ca/Al compared to the neat PCL. In case of ROP-PCL prepared in the presence of IL-modified LDH, significant increase of *T_d5%_* probably corresponds to much larger crystallites (2.8-fold) present in this sample compared to those in the neat PCL. Moreover, phosphonium compounds are known as efficient thermal stabilizers for polymer materials [29,30]. Thus, the phosphonium-based IL (IL-D) bonded on the filler surface acts as the thermal stabilizer of PCL matrix in the case of ROP-PCL + C-Ca/Al-D nanocomposite.

In case of nanocomposites prepared by melt-blending, decrease of *T_d5%_* is presumably related to the partial thermal degradation of PCL during sample preparation. Therefore, the M_n_ values of prepared PCL-LDH nanocomposites are lower than M_n_ of the initial MB-PCL (Table 3, Figure S5). Although the large PCL crystallites are formed in the samples containing LDHs, poor filler distribution in MB-PCL + C-Ca/Al (Figure S6 does not improve the overall thermal stability of MB-PCL + C-Ca/Al. Moreover, the brownish color of MB-PCL + C-Ca/Al-D material (Figure S6) reflects chemo-mechanical degradation of the IL-functionalized LDH, which probably originated from high shear forces applied during the melt-blending.

Contact angle measurements determined by the sessile drop method showed that the use of hydrophilic non-modified C-Ca/Al LDH decreases the water contact angle, *θ_H2O_* (Table 2). The nanocomposite films with the C-Ca/Al filler are thus more hydrophilic than the neat PCL films. Contrary to that, the incorporation of IL-modified filler (C-Ca/Al-D) decreases the *θ_H2O_* value only in the case of melt-blended nanocomposite films, while the in situ prepared C-Ca/Al-D nanocomposite exhibits the same hydrophilicity as the neat PCL film (Table 2). It is known that the hydrophobic nature of phosphonium IL bearing long alkyl chains [31] used for the modification of hydrophilic LDH particles decreases overall polarity of PCL/LDH nanocomposites only when the filler is homogenously dispersed in the whole volume of PCL matrix [26]. The contact angle thus indirectly indicates that the in-situ prepared nanocomposite (ROP-PCL + C-Ca/Al-D) contains more homogenously dispersed LDH fillers than the nanocomposite prepared by melt-blending (MB-PCL + C-Ca/Al-D). The results further show the decreased hydrophilicity of the nanocomposites containing C-Ca/Al-D in comparison to the nanocomposites with C-Ca/Al, which indicates the presence of IL-D on the filler surface. As a consequence, the surface-immobilized IL-D molecules can be highly accessible for compatibilization of LDH fillers with the hydrophobic PCL matrix.

Further evidence that the surface IL-D functionalization of nanoparticles improves their compatibility with the PCL matrix is seen from mechanical testing. Table 3 summarizes the results of uniaxial tensile properties of the prepared PCL nanocomposite films.

Generally, the LDH addition leads to an improvement in material stiffness (the increased Young modulus, E) resulting from enhanced rigidity, which is induced by the incorporation of nanofiller to polymer matrix [4]. Herein, the Young’s modulus of the PCL nanocomposite with non-modified C-Ca/Al (MB-PCL + C-Ca/Al) prepared by melt-blending was decreased by adding 1 wt % of LDH nanofillers. Moreover, this material exhibited the decreased values of tensile strength (σ_max_) as well as maximal elongation (ε_max_), which indicates formation of agglomerates (even macroscopically visible—see the photos in Supplementary Figure S6), overall heterogeneous filler distribution and low compatibility of the non-modified LDH. Incorporation of the IL-modified LDH via melt-blending technique (MB-PCL + C-Ca/Al-D sample) led to improved nanofiller dispersion within PCL matrix as indicated from the slightly increased *E* values (+5%), the macroscopic photos (Supplementary Figure S6) and the increased crystallinity (Table 2). However, the σ_max_ and ε_max_ values of this material are comparable to those of the neat PCL, which indicates that interfacial nanofiller-polymer interactions are less intensive. Moreover, as discussed above, IL-functionalized LDH degraded during the melt-blending, which can further decrease the mechanical properties of the prepared MB-PCL + C-Ca/Al-D material.

Contrary to that, the Young moduli of the in situ ROP prepared nanocomposites are significantly improved (+21% for ROP-PCL + C-Ca/Al and +24% for ROP-PCL + C-Ca/Al-D, Table 3), which indicates much better nanofiller dispersion in PCL matrix compared to the nanocomposites prepared by melt-blending. Moreover, it is important to notice that the mechanical properties of the in situ prepared nanocomposites are significantly affected by different molecular weights of the synthesized PCL (Table 3, Figure S5). The M_n_ values of the in situ prepared ROP-PCL nanocomposites are lower than M_n_ of the pure ROP-PCL due to the initiating capability of water molecules adsorbed on the LDH nanoparticles (for more details–see our last paper [19]). Generally, mechanical properties increase with the increased length of polyester chain [32,33]. Although both in situ prepared nanocomposites exhibited the lower average molecular weight (Table 3, Figure S5) and the lower amount of the crystalline phase (Table 2) than the neat ROP-PCL, the Young moduli of these nanocomposites increased significantly (Table 3). This phenomenon has to be a consequence of the increased rigidity induced by the nanofillers, which are homogeneously dispersed within the PCL matrix. Moreover, although the average molecular weight of the ROP-PCL + C-Ca/Al-D nanocomposite was the lowest, this material exhibited the increased elongation at break and the stiffness similar to the neat PCL, which had much higher M_n_. The IL-D ability to act as a surfactant and compatibilizer providing the increased C-Ca/Al-D nanofiller/PCL affinity during the in situ microwave-assisted ROP of *ε*CL was thus clearly demonstrated.

### 3.3. Gas/vapor Properties of PCL-LDH Nanocomposites

**Figure 4:**
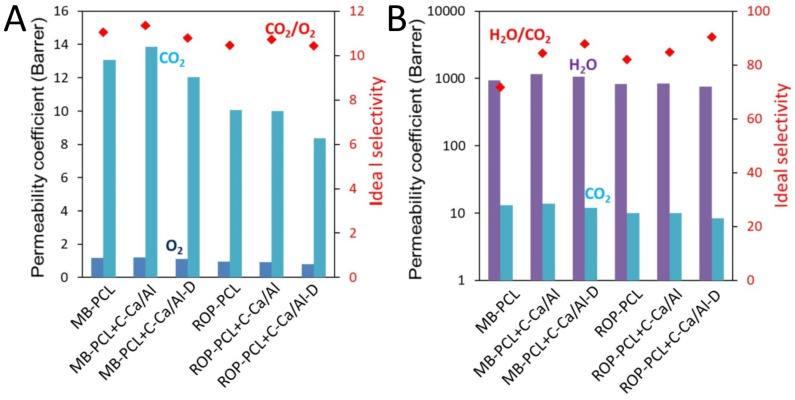
Carbon dioxide, oxygen and water vapor permeability and corresponding ideal selectivity of prepared materials: CO_2_/O_2_ (**A**) and H_2_O/CO_2_ (**B**).

Considering the potential application of the developed PCL nanocomposite films as packaging materials, their gas/water vapor barrier properties are highly important. Moreover, gas and vapor molecules can act as probes which can reveal some interesting (semi-quantitative) information about the inner structure and physicochemical behavior of the material (free volume, polarity, chain packing and flexibility, etc.). Therefore, permeability (Figure 4, Table S1), diffusion (Table S2) and solubility (Table S3) coefficients as well as the corresponding ideal selectivities for CO_2_, O_2_, and water vapor were determined on the prepared materials.

The permeability coefficients (*P*) of all tested PCL-LDH films increased in the order of oxygen < carbon dioxide << water vapor (Table S1). The permeability measurements were carried out at temperatures above *T*_g_ of the PCL nanocomposites. The materials thus were in the rubbery state and under those conditions, permeation, solubility and diffusion mechanisms were similar to gas transports in liquids. Nanocomposite films based on MB-PCL matrix had generally higher *P* then the ROP-PCL series. This can be purely attributed to the differences of the material properties between MB-PCL and ROP-PCL. Consistently lower diffusion coefficient (*D*) for ROP-PCL based materials indicates lower fractional free volume (part of the polymer free volume which is accessible for the gas molecules) or in other words, these materials exhibit more intense macromolecule chain packing. The crystallinity of the polymers is usually a significant factor influencing the transport properties (higher crystallinity means lower permeability), but in our case this effect is less apparent. Crystallinity is significantly higher and *P* lower for the neat ROP-PCL compared to MB-PCL, but after filler addition the trend is not so obvious (e.g., ROP-PCL + C-Ca/Al has contrary lowest crystallinity of all materials).

Since the permeability (*P*) of penetrant molecules is proportional to *D* and *S*, the more prevailing process is assessed on the basis of diffusion and solubility selectivities. In MB-PCL and ROP-PCL films the permeabilities of gas/water vapor were driven by the solubility rather than by diffusion (much higher solubility selectivities than the diffusion selectivities) (Tables S2 and S3). Solubility contribution to permeability of a membrane is correlated with the amount and intensity of interactions between molecules of gas/vapor and polymer matrix [34,35]. High solubility coefficients of polar molecules (CO_2_ and H_2_O) clearly indicate the presence of molecular interactions between these polar penetrants and relatively high polar PCL macromolecules due to the presence of ester linkages. Contrary to that, the solubility coefficient of O_2_ is much lower, which further results in significantly slower permeation of neutral O_2_ molecules through PCL materials as compared to CO_2_ and water.

Because of the relatively low weight content (1.0 wt %) of the fillers, their influence on the gas transport properties was very mild. Nevertheless, it can be clearly seen that films containing 1.0 wt % C-Ca/Al had higher *P*, *D* and even *S* compared to films with 1.0 wt % C-Ca/Al-D. This clearly indicates that the IL-modified C-Ca/Al-D filler is better dispersed in the PCL matrix than the non-modified C-Ca/Al.

High water vapor permeabilities (WVP) of the neat PCL (Figure 4) is one of main drawbacks, which limits the use of PCL films for food packaging. In our case, the addition of very low amount (1.0 wt %) of IL-functionalized LDH (Figure 4, Table S1) showed the relative WVP (expressed as a ratio of WVP of the nanocomposites and WVP of neat polymer) around 0.9, which indicates that IL-LDH can decrease WVP of PCL nanocomposites more efficiently than other 2D nanofillers, e.g., organically modified MMT (the similar WVP reduction was reached using a five-fold higher MMT content) [36]. Unfortunately, the preparation of ROP-PCL nanocomposites with the higher C-Ca/Al-D loadings (e.g., 5.0 wt %), which would probably further improve WVP, still remains a challenging issue as the increased C-Ca/Al-D content results in low molecular weight PCL with unsatisfactory mechanical properties. For this purpose, the application of different types of LDH having a lower affinity to water than the Ca/Al-based LDH can be useful, e.g., fully or partially calcinated Zn/Al-based LDH, which in a hydrated form contains ca. six times lower water content than the hydrated Ca/Al LDH [37].

## 4. Conclusions

In this work, we successfully prepared crystalline calcinated Ca/Al LDH nanoparticles with a relatively high content (18 wt %) of surface immobilized phosphonium ionic liquid (IL-D). Due to thermal treatment (calcination) and surface organophilization by IL-D, the synthesized LDH exhibited low water content (1.5 wt %), reduced hydrophilicity, as well as improved thermal stability compared to pristine LDH. Altogether, these beneficial properties make thus modified LDH nanoparticles suitable to be applied as functional nanofillers in water-sensitive polymer matrices.

Herein, the LDH nanoparticles with immobilized IL-D were applied for the preparation of polycaprolactone (PCL) nanocomposite films. Comparison of two ways of nanocomposite preparation clearly indicated that the microwave-assisted in situ ring opening polymerization (ROP) of *ε*-caprolactone (*ε*CL) in the presence of 1 wt % IL-functionalized LDH led to homogenous nanofiller dispersion in the PCL matrix promoting formation of large PCL crystallites, which positively affected mechanical and barrier properties of the final nanomaterial. The surface bonded IL-D thus acted as a nanofiller surfactant, compatibilizer, as well as stabilizer improving the thermal resistance of PCL/LDH nanocomposites. Contrary to that, the melt-blending technique led to poor nanofiller dispersion and partial IL-D degradation during the processing and production of PCL nanocomposites with decreased mechanical and barrier properties.

Despite the fact that the water affinity of the synthesized IL-functionalized calcinated LDH nanoparticles was highly reduced compared to pristine LDH, the preparation of PCL nanocomposite films with higher nanofiller loadings is still a challenging issue for this calcium/aluminum type of LDH. Therefore, the synthesis of different types of LDH (e.g., Zn/AL-based) with low water affinity is planned for the future.

## Figures and Tables

**Table 1:** Neat PCL and PCL-LDH nanocomposites prepared in this work.

Sample ID	Sample Description
MB-PCL	Neat commercial PCL
MB-PCL + C-Ca/Al	Commercial PCL containing 1.0 wt % of C-Ca/Al LDH prepared by melt blending technique
MB-PCL + C-Ca/Al-D	Commercial PCL containing 1.0 wt % of C-Ca/Al-D LDH prepared by melt blending technique
ROP-PCL	PCL prepared by in situ ROP under microwave irradiation
ROP-PCL + C-Ca/Al	PCL prepared by in situ ROP under microwave irradiation in the presence of1.0 wt % of C-Ca/Al LDH
ROP-PCL + C-Ca/Al-D	PCL prepared by in situ ROP under microwave irradiation in the presence of 1.0 wt % of C-Ca/Al-D LDH

**Table 2:** Crystalline domain sizes, thermal behavior, water contact angle of PCL-LDH nanocomposite and neat PCL films.

Sample	*D*_(110)_ [Å]	*T_α_* [°C]	*T_m_* [°C]	Δ*H_m_* [J/g]	*X_c_* [%]	*T_d5%_* [°C]	*θ_H2O_* [^o^]
MB-PCL	73	−54.1	55.9	54.5	42.3	354.0	90 ± 3
MB-PCL + C-Ca/Al	161	−54.4	53.6	59.6	42.8	307.1	81 ± 4
MB-PCL + C-Ca/Al-D	179	−55.6	55.9	64.3	46.2	318.1	82 ± 3
ROP-PCL	86	−57.5	53.6	65.1	46.8	304.3	81 ± 1
ROP-PCL + C-Ca/Al	188	−56.8	55.4	51.2	37.2	308.0	75 ± 3
ROP-PCL + C-Ca/Al-D	245	−55.4	56.9	59.0	42.9	318.7	79 ± 2

*D*_(110)_—size of crystalline domains, *T_α_*—main transition temperature from DMTA; *T_m_*—(onset) melting temperature, Δ*H_m_*—melting enthalpy and *X_c_*—crystallinity from DSC, 2nd heating run; *T_d5%_*—temperature of 5% weight loss of the sample from TGA; *θ_H2O_*—contact angle.

**Table 3:** Average molecular weight (*M*_n_), dispersity (Ð) and tensile properties of neat PCL and PCL nanocomposites.

Sample	M_n_∙10^3^ [g/mol]	Ð	Young Modulus, E [MPa]	Tensile Strength, σ_max_ [MPa]	Elongation at Break, ε_max_ [%]
MB-PCL	126	1.22	443.0 ± 9.1	32.6 ± 2.5	424 ± 22.4
MB-PCL + C-Ca/Al	117	1.28	434.0 ± 7.6	28.7 ± 5.2	382 ± 59.2
MB-PCL + C-Ca/Al-D	120	1.31	465.0 ± 10.4	31.7 ± 1.8	419 ± 18.5
ROP-PCL	99	1.43	463.0 ± 12.6	28.2 ± 1.4	390 ± 12.7
ROP-PCL + C-Ca/Al	79	1.42	560.0 ± 7.1	17.7 ± 13.2	353 ± 77.0
ROP-PCL + C-Ca/Al-D	61	1.59	572.0 ± 26.2	25.4 ± 2.0	423 ± 23.0

## Supplementary Materials

The following are available online at https://www.mdpi.com/2079-4991/10/5/969/s1, Figure S1: ATR-FTIR spectra (A), XRD/WAXS patterns (B) and SEM micrographs (C) of synthesized LDH: (a) pristine LDH (Ca/Al), (b) calcinated LDH (C-Ca/Al) and (c) calcinated LDH modified with ionic liquid (C-Ca/Al-D); Figure S2: DSC curves of neat PCL and PCL-LDH nanocomposite samples prepared by melt-intercalation (A) and in situ microwave-assisted ring opening polymerization (B); Figure S3: Storage modulus, loss modulus and loss tangent of neat PCL and PCL-LDH nanocomposite samples (DMTA); Figure S4: TGA curves of neat PCL and PCL-LDH nanocomposite samples; Figure S5: GPC curves of neat PCL and PCL-LDH nanocomposite samples; Figure S6: Photos of prepared neat PCL and PCL/LDH nanocomposite films (thickness of 0.2 mm); Table S1: Permeability coefficients for O_2_, CO_2_ and water vapor and corresponding ideal selectivities; Table S2: Diffusion coefficients for O_2_, CO_2_ and water vapor and corresponding diffusivity selectivities; Table S3: Solubility coefficients for O_2_, CO_2_ and water vapor and corresponding solubility selectivities.
