# Supplementary material for: Effects of Immobilized Ionic Liquid on Properties of Biodegradable Polycaprolactone/LDH Nanocomposites Prepared by In Situ Polymerization and Melt-Blending Techniques
Source: Nanomaterials (Basel). 2020 May 18;10(5):969. doi: 10.3390/nano10050969 (PMC7712423; doi:10.3390/nano10050969)
Supplement: Supplementary file 1 [file nanomaterials-10-00969-s001.pdf]

Supporting information for:

Effects of immobilized ionic liquid on properties of biodegradable polycaprolactone/LDH nanocomposites prepared by *in-situ* polymerization and melt-blending techniques

Sonia Bujok, Jiří Hodan, Hynek Beneš*

Institute of Macromolecular Chemistry, CAS, Heyrovského nám. 2, 162 06 Prague 6, Czech Republic.

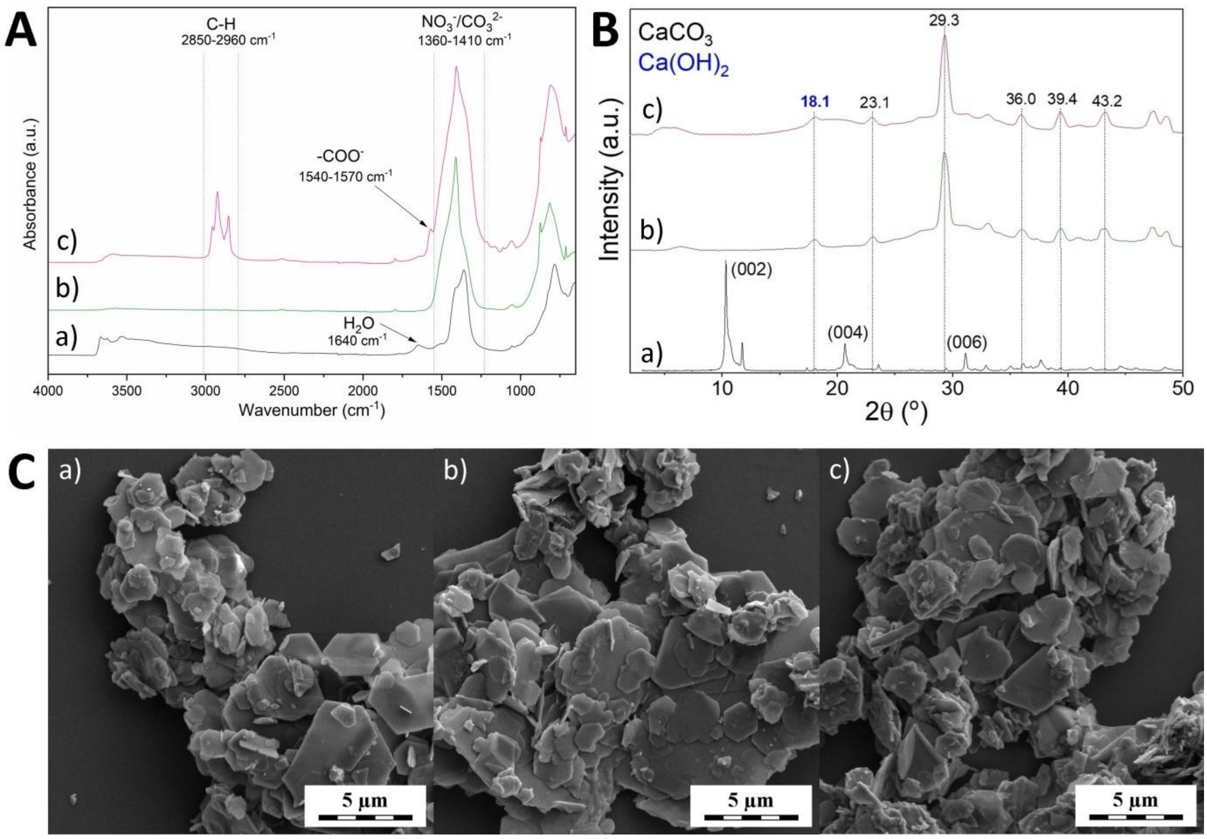

Figure S1. ATR-FTIR spectra (A), XRD/WAXS patterns (B) and SEM micrographs (C) of synthesized LDH: a) pristine LDH (Ca/Al), b) calcinated LDH (C-Ca/Al) and c) calcinated LDH modified with ionic liquid (C-Ca/Al-D).

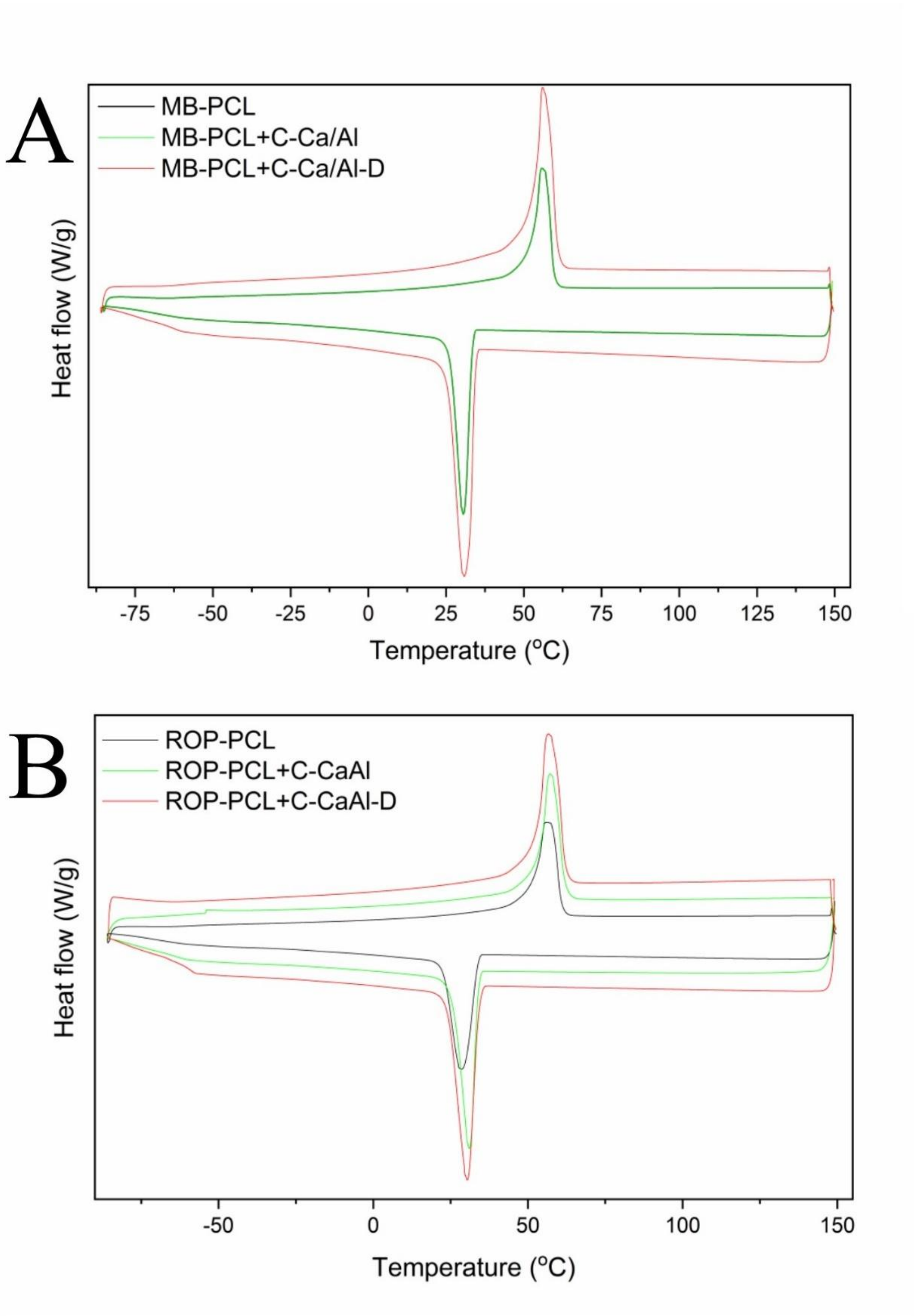

Figure S2. DSC curves of neat PCL and PCL/LDH nanocomposite samples prepared by melt-blending (A) and *in-situ* microwave-assisted ring opening polymerization (B).

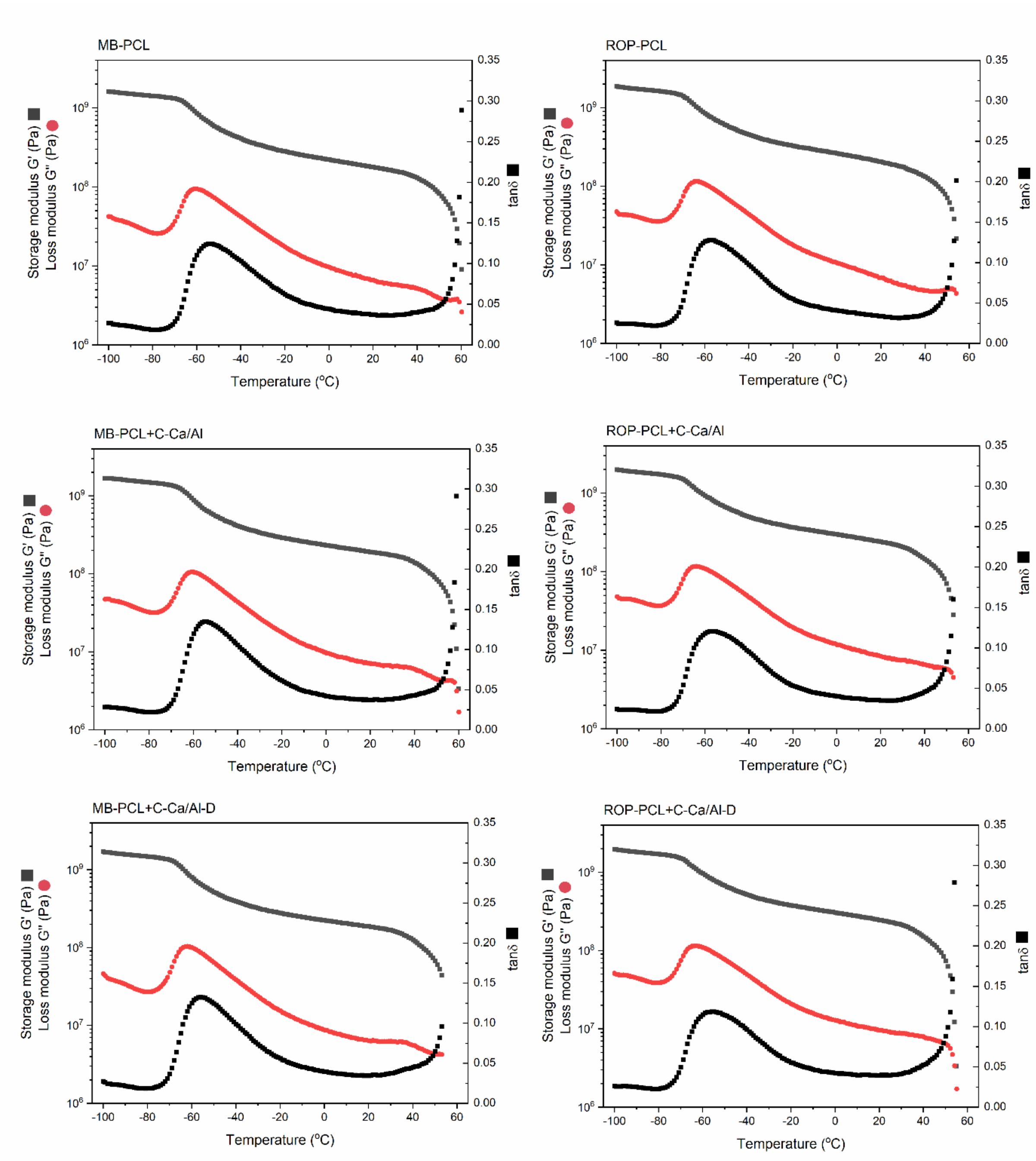

Figure S3. Storage modulus, loss modulus and loss tangent of neat PCL and PCL/LDH nanocomposite samples (DMTA).

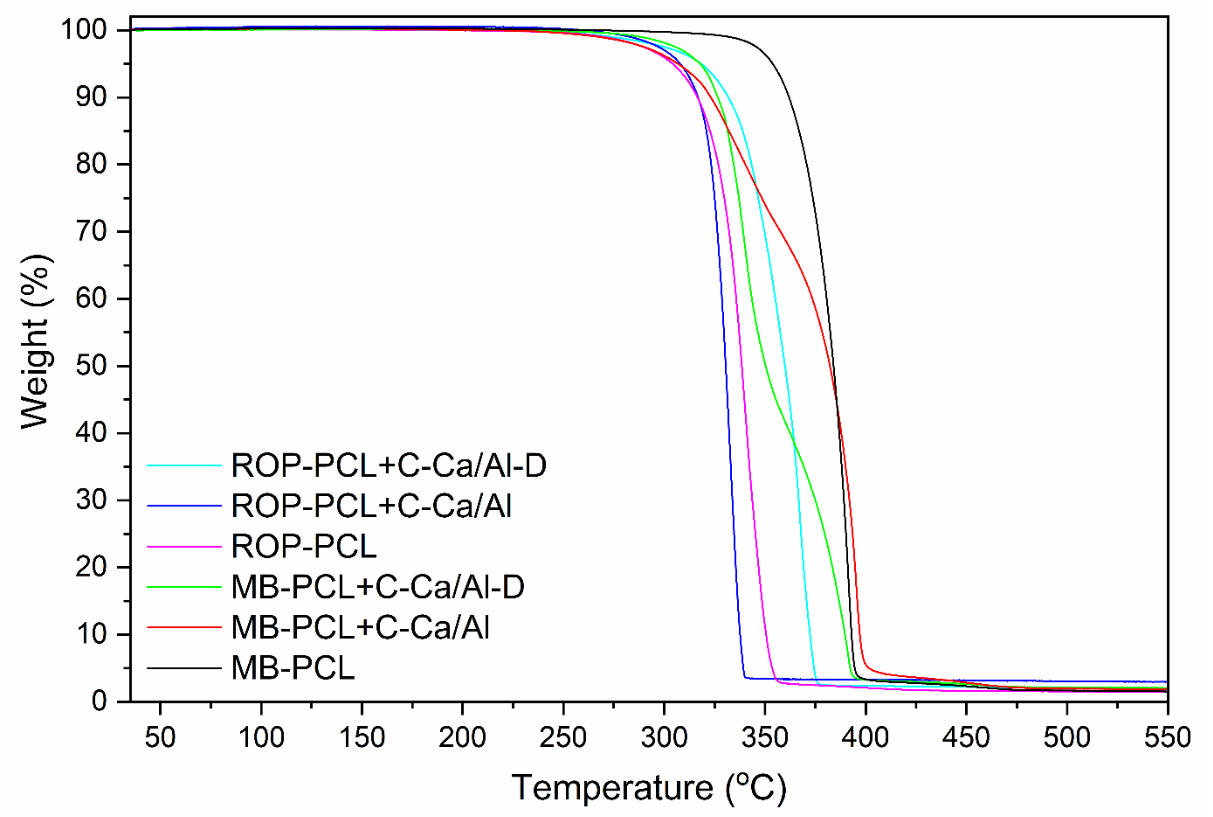

Figure S4. TGA curves of neat PCL and PCL/LDH nanocomposite samples.

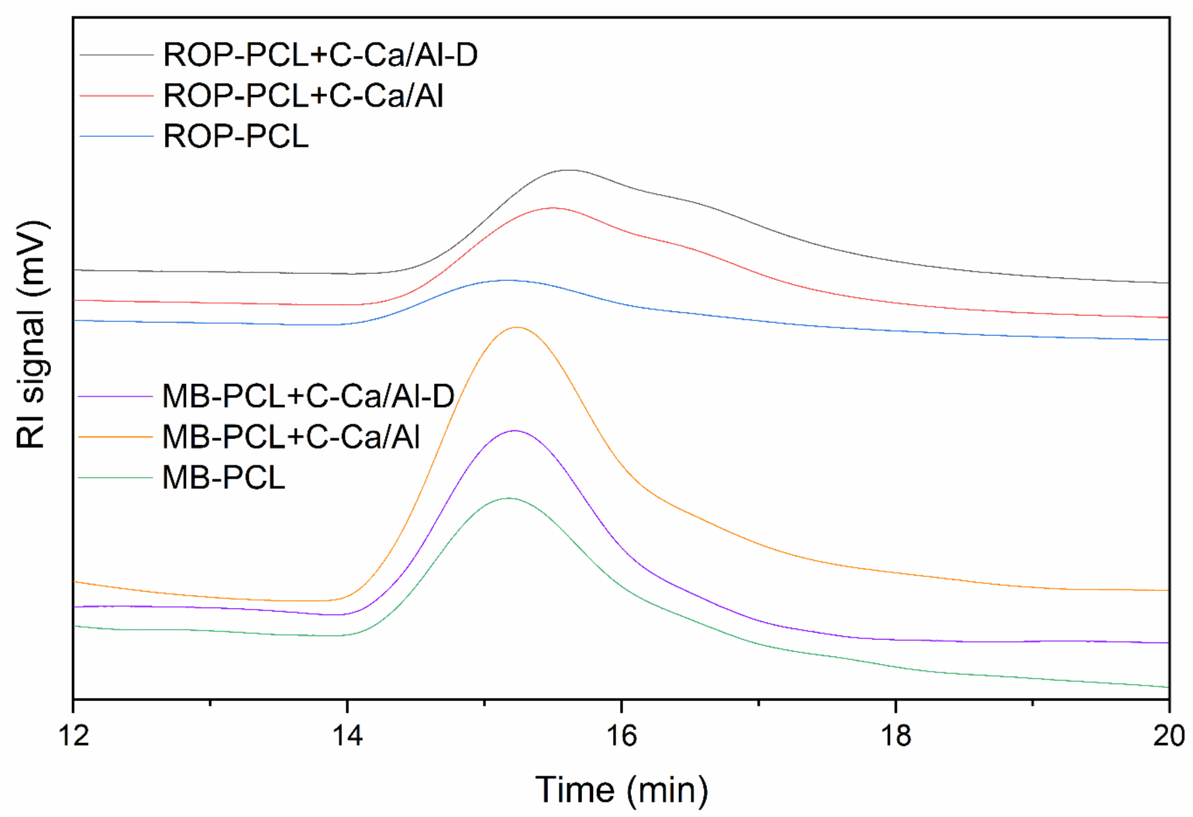

Figure S5. GPC curves of neat PCL and PCL/LDH nanocomposite samples.

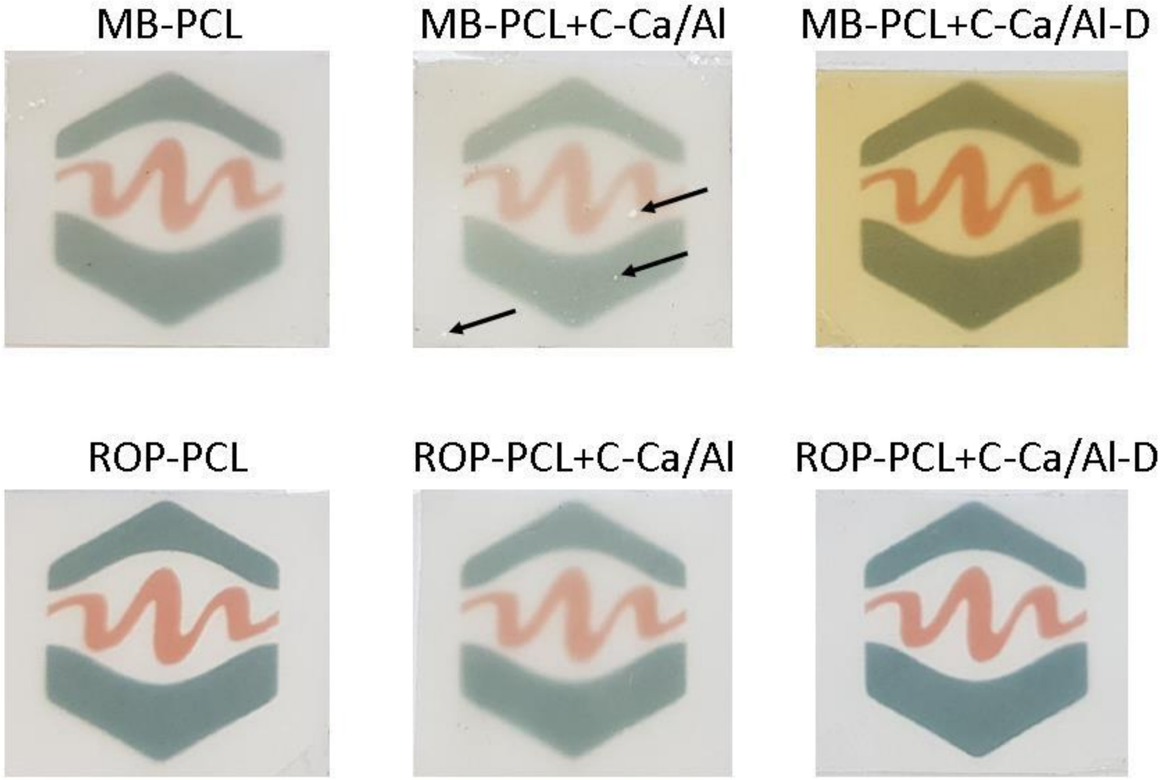

Figure S6. Photos of prepared neat PCL and PCL/LDH nanocomposite films (thickness of 0.2 mm).

Table S1. Permeability coefficients for O_2, CO_2 and water vapor and corresponding ideal selectivities.

Material	*P* (Barrer)[1]			Ideal selectivity		
	O_2	CO_2	H_2O	CO_2/O_2	H_2O/O_2	H_2O/CO_2
MB-PCL	1.18	13.1	939	11.0	793	72
MB-PCL+C-Ca/Al	1.22	13.9	1171	11.4	959	84
MB-PCL+C-Ca/Al-D	1.12	12.0	1058	10.8	948	88
ROP-PCL	0.96	10.1	826	10.5	858	82
ROP-PCL+C-Ca/Al	0.93	10.0	847	10.7	909	85
ROP-PCL+C-Ca/Al-D	0.80	8.35	755	10.5	946	90

[1]Barrer = 1 $\cdot 10^{-10}$ cm^3 (STP)cm $\cdot cm^{-2}$ s^{-1} $cmHg^{-1}$ = 3.3539 $\cdot 10^{-16}$ mol s^{-1} $\cdot m^{-1}$ $\cdot Pa^{-1}$

Table S2. Diffusion coefficients for O_2, CO_2 and water vapor and corresponding diffusivity selectivities.

Material	D ×10^8 (cm^2/s)			Diffusivity selectivity		
	O_2	CO_2	H_2O	CO_2/O_2	H_2O/O_2	H_2O/CO_2
MB-PCL	27	13.5	7.6	0.50	0.28	0.56
MB-PCL+C-Ca/Al	29	13.7	6.3	0.47	0.22	0.46
MB-PCL+C-Ca/Al-D	28	12.8	7.1	0.45	0.25	0.56
ROP-PCL	16	11.4	4.9	0.71	0.31	0.43
ROP-PCL +C-Ca/Al	26	11.1	4.3	0.43	0.16	0.38
ROP-PCL +C-Ca/Al-D	25	9.91	3.5	0.39	0.14	0.35

Table S3. Solubility coefficients for O_2, CO_2 and water vapor and corresponding solubility selectivities.

Material	*S*×10^2 [cm^3(STP)/(cm^3 $\cdot$cmHg)]			Solubility selectivity		
	O_2	CO_2	H_2O	CO_2/O_2	H_2O/O_2	H_2O/CO_2
MB-PCL	14.6	326	42 000	22.3	2900	129
MB-PCL+C-Ca/Al	14.1	340	63 000	24.2	4500	185
MB-PCL+C-Ca/Al-D	13.3	317	50 000	23.9	3800	158
ROP-PCL	20.2	298	57 000	14.7	2900	191
ROP-PCL +C-Ca/Al	12.1	302	67 000	25.0	5600	222
ROP-PCL +C-Ca/Al-D	10.5	283	73 000	26.9	7000	258
